# Quality of DNA extracted from freshwater fish scales and mucus and its application in genetic diversity studies of *Perca fluviatilis* and *Rutilus rutilus*

**DOI:** 10.1093/biomethods/bpad022

**Published:** 2023-09-28

**Authors:** Ieva Ignatavičienė, Adomas Ragauskas, Vytautas Rakauskas, Dalius Butkauskas

**Affiliations:** Nature Research Centre, Vilnius 08412, Lithuania; Nature Research Centre, Vilnius 08412, Lithuania; Nature Research Centre, Vilnius 08412, Lithuania; Nature Research Centre, Vilnius 08412, Lithuania

**Keywords:** fish scales, skin mucus, swabbing, DNA extraction, mtDNA D-loop, ATP6, *P. fluviatilis*, *R. rutilus*

## Abstract

Studies on genetic diversity require biological material containing a reliable source of DNA that can be extracted and analyzed. Recently, non-invasive sampling has become a preferred sampling method of biological material. The suitability of a less invasive approach that involves obtaining samples by swabbing the fish skin (including live, non-anesthetized fish) should be considered. In this study, we compared the efficiency of DNA extraction, amplification, and sequencing of mtDNA fragments of two fish species *Perca fluviatilis* and *Rutilus rutilus* based on DNA collected from the scales and mucus using the modified Aljanabi and Martinez method. The results revealed a higher quality of DNA extracted from the mucus; however, the mean DNA concentration obtained from the scales of both fish species was higher. We verified the method suitable for amplification and sequencing of mtDNA fragments of both fish species using newly designed markers (D-loop, ATP6) and examined the potential risk of intraspecific cross-contamination. The DNA sequence alignment analysis revealed identical sequences attributed to the same individual when DNA, extracted from two different sources (scales and mucus), was used. We demonstrated that the quantity and quality of DNA extracted from the scales and mucus using the proposed method were high enough to carry out genetic diversity studies based on sampling of live fish with the possibility to release it after collecting samples.

## Introduction

To analyze the genetic diversity of freshwater fish, different biological materials such as muscle, blood, or fin are used to extract DNA [1]. Recently, non-invasive sampling has become a preferred choice. However, using non-invasive sampling, its application becomes more challenging due to the low yield and/or low quality of DNA obtained from such sources as mucus or scales [2]. Some authors consider that methods based on DNA extraction from fish scales should be attributed to invasive sampling as scales need to be physically removed from the fish [3]. In other studies, scale sample collection was identified as non-invasive [4,5] since the removal of scales might cause an acute stress responsein the fish; however, long-term studies suggest that they have no significant effect on fish health [6]. Scale samples have been found to yield enough DNA to be used in various genetic studies [7].

Fish mucus is produced by goblet cells. It is a viscous colloid substance that contains water, antibacterial enzymes, and high molecular weight thread-like glycoproteins called mucins [8]. The external mucus (covering skin and gills) is the main surface of exchange between fish and their surrounding environment. It represents an important protective barrier against pathogens since it reduces colonization by pathogenic organisms (bacteria, viruses, or eukaryotic organisms) [9]. The mucus has been studiedby different authors to analyze the microbiota [10] or fish–parasite system [11] after it was obtained by the so-called “scraping” [11, 12], “swabbing/wiping” [13, 14], or “massaging of fish in a plastic bag” [15]. Each method was associated with its set of challenges. The histological analysis of skin regions after mucus had been sampled by either absorption, wiping, or scraping showed that while the scraping method was the most invasive to the skin epidermis, the “swabbing/wiping” method was the least invasive [16]. Swabbing appears to be less invasive than DNA extracted from the muscle or fin; since it requires no anesthetic or the need to remove a tissue, it should be considered as the most efficient non-invasive method. So far, the DNA extraction method from the mucus has been still questionable due to DNA yield, polymerase chain reaction (PCR) amplification, and the potential for cross-contamination [17, 18].

Mitochondrial (mtDNA) markers have been widely used to evaluate the genetic diversity and characteristics of the populations, including different fish species. mtDNA is relatively easy to amplify, it is not duplicated, typically nonrecombining, nearly neutral, and highly variable between and within species [19]. Thecontrol region of mtDNA (D-loop) is the region with the largestvariation in the whole mtDNA genome sequence and length. Its evolution rate is 5–10 times higher than that of other sequences, resulting in more mutations, such as base replacement, insertion, or deletion, and the changing number of tandem repeats. The nucleotide substitutions in this sequence can be studied to explore the genetic relationship between species or individuals [20]. The conservative sequences of Cytochrome b [21, 22] and ATP8/6 [23] genes also have been widely applied to analyze phylogenetics and genetic relationships based on inter- and intra-specific genetic variability of fish populations.

In this study, we develop a protocol of DNA extraction from the freshwater fish scale and mucus of the Eurasian perch (*Perca fluviatilis*) and the Common roach (*Rutilus rutilus*) based on the modified Aljanabi and Martinez [24] method. Our study provides evidence confirming that high yield and quality DNA obtained from the mucus and scale could be successfully used to amplify and sequence different mtDNA fragments.

## Methods

### Sample collection and DNA extraction

A total of 10 (average 64.3 ± 9.2 g) perch and 10 (average 51.6 ± 6.7 g) roach specimens were collected from Lake Drūkšiai (55°38′45.7″N 26°35′53.3″E on 11 October 2022) using the appropriate permit issued by the State Fish Monitoring Program. All fish specimens were caught by the net and transferred to the Laboratory of Molecular Ecology, Nature Research Centre (Vilnius, Lithuania) and placed in a separate bag to avoid possible DNA contamination, especially via mucus of different or the same fish species. Before DNA extraction, fish specimens were stored at −20°C. Total DNA was extracted from the mucus and scales of specimens of both species using a modified version of Aljanabi and Martinez [24]. Before DNA extraction, fish specimens from the freezer were thawed. Approximately 50 mg of scales were separated from each fish using a sharp knife and stored in 400 µl of sterile salt homogenizing buffer (0.4 M NaCl, 10 mM Tris–HCl pH 8.0, and 2 mM EDTA pH 8.0). In the case of mucus, the head area of each fish was wiped with a cotton swab. To obtain enough material for DNA extraction, the mucus collection procedure from each specimen was repeated six times in total. Then the used cotton swab was placed in 400 µl of sterile salt homogenizing buffer for 15 min. Then it was removed and the homogenizing solution containing mucus was well mixed. An aliquot of 40 µl of 20% SDS (2% final concentration) and 8 µl of 20 mg/ml proteinase K (400 µg/ml final concentration) were added and mixed well. The same procedure was applied for scales and lysates of mucus, and scales were prepared for incubation. The lysate of each sample was incubated at 65°C for 1 h with intermittent vortexing at every 10 min intervals. After 1 h of incubation, 300 µl of 6 M NaCl (NaCl-saturated H_2_O) was added to each sample. Samples were vortexed for 30 s at maximum speed, and tubes were spun down for 30 min at 10 000 g. The supernatant was transferred to fresh tubes. An equal amount of isopropanol was added to each sample, mixed well, and the samples were incubated at −20°C for 1 h. Then the samples were centrifuged for 20 min, 4°C, at 10 000 g. The pellet was washed with 70% ethanol, dried, and finally resuspended in 100–300 µl sterile dH_2_O. Two microliters of purified DNA solution were used to measure the quality and quantity of DNA (ng/μl) (A260/A280) with the help of NanoPhotometer P330 (IMPLEN, Germany). Each sample of DNA obtained was analyzed on 1.5% agarose gel in 1X Tris–acetate–EDTA (TAE) buffer using Thermo Scientific Gene-Ruler DNA ladder (Thermo Fisher Scientific, Vilnius, Lithuania) and visualized by ethidium bromide staining. DNA extracts were frozen at −20°C until further use.

### PCR conditions and sequencing

When setting up PCR experiments, primers previously used for amplification and sequencing of mtDNA *ATP6* and *cox3* gene fragments and *D-loop* region of the perch [25] and newly created primer pairs designed to amplify *ATP6* gene fragment and *D-loop* region of the roach were used. Detailed information about the primers and PCRs conditions are presented in Table 1. The PCR reactions were prepared in 10 µl of final solution volume containing 2 µl of DNA (50 ng/µl), 1 μl of forward and 1 μl of reverse primer (10 μM), 5 µl of DreamTaq DNA polymerase mix (Thermo Fisher Scientific Baltics, Lithuania), 1 µl of nuclease-free water in Eppendorf Mastercycler thermocycler. Amplified products were analyzed using electrophoresis in 1.5% agarose gel in 1X TAE buffer using Thermo Scientific Gene-Ruler DNA ladder (Thermo Fisher Scientific, Vilnius, Lithuania) and visualized by ethidium bromide staining. The PCR products were purified with exonuclease I and FastAP Thermosensitive Alkaline Phosphatase (Thermo Fisher Scientific Baltics, Vilnius, Lithuania). Sequencing reactions were performed using the Big-Dye Terminator version 3.1 Cycle Sequencing Kit and the 3500 Genetic Analyzer (Applied Biosystems, Foster City, California, USA) according to the manufacturer’s recommendations. PCR products were sequenced directly using the PCR forward and reverse primers.

**Table 1.** PCR conditions for amplification of selected mtDNA fragments of *P. fluviatilis* and *R. rutilus* using DNA extracted from fish mucus or scales.

Species	mtDNA region	Primer pairs	PCR conditions	Products, bp
*P. fluviatilis*	*ATP6* and *cox3*	**ATP-PCR-F:** 5′-CCCTAACGAGCCTACATCCC-3′	2 min at 95°C, 35 cycles (30 s at 94°C, 45 s at 52°C, 45 s at 72°C), 5 min at 72°C	888
		**ATP-PCR-R:** 5′-TGTAAGAGGTCAAGGGCTGG-3′		
	*D-loop*	**HV2:** 5′-TTCCCCGGTCTTGTAAACC-3′	5 min at 96°C, 30 cycles (1 min at 96°C, 1 min at 54°C, 2 min at 72°C), 5 min at 72°C. [27]	550
		**CSB-D:** 5′-GGAACCAAATGCCAGGAA-3′ [26]		
*R. rutilus*	*ATP6*	**ATP6_L:** 5′-TCCAACTCCACCATCTCGTT-3′	2 min at 94°C, 35 cycles (30 s at 94°C, 45 s at 52°C, 45 s at 72°C), 5 min at 72°C.	520
		**ATP6_R:** 5′-GCCTGGATTATGGCTACTGC-3′		
	*D-loop*	**Rut_2F:** 5′-GTTTCGGGGTTTGACAAAGA-3′	2 min at 95°C, 35 cycles (1 min at 94°C, 45 s at 59°C, 45 s at 72°C), 5 min at 72°C.	590
		**Rut_2R:** 5′-AGGTCAGGACCATGCCTTTA-3′		

### Molecular data analysis

The mean DNA concentration was analyzed for significance using a two-tailed unpaired Student’s *t*-test [28] and presented in Fig. 2. The *P *< 0.05 was considered significantly statistically different. Sequenced mtDNA fragments were aligned using MUSCLE [29] option in MEGA-X [30]. Nucleotide substitutions determined in each mtDNA fragment after comparing the newly obtained sequences with reference sequences (LC495488.1 and AP005995.1 accession numbers represent perch *ATP6* and *cox3,* and *D-loop* fragments, respectively; OM736800.1 and DQ455036.1 accession numbers represent roach *ATP6* and *D-loop* fragments, respectively) selected from GenBank are shown in Table 2.

**Table 2.** Nucleotide substitutions of mtDNA *ATP6* and *D-loop* regions of perch and roach.

*Perca fluviatilis*						*R. rutilus*					
mtDNA region	ATP6 and cox3		D-loop			mtDNA region	ATP6	D-loop			
GenBank accession number	LC495488.1		AP005995.1			GenBank accession number	OM736800.1	DQ455036.1			
Position	8430	8463	15849	15658	15659	Position	8666	548	800	831-832	910
Sample Code	C	A	A	G	C	Sample	T	T	T	-	G
1P-M			G			1R-M		C			
1P-S			G			1R-S		C			
2P-M			G	A	A	2R-M		C	C		
2P-S			G	A	A	2R-S		C	C		
3P-M			G	A	A	3R-M		C			
3P-S			G	A	A	3R-S		C			
4P-M	T					4R-M		C	C		
4P-S	T					4R-S		C	C		
5P-M			G	A	A	5R-M				T	
5P-S			G	A	A	5R-S				T	
6P-M		G	G			6R-M					
6P-S		G	G			6R-S					
7P-M	T					7R-M					
7P-S	T					7R-S					
8P-M	T					8R-M	C				
8P-S	T					8R-S	C				
9P-M			G	A	A	9R-M		C			A
9P-S			G	A	A	9R-S		C			A
10P-M	T					10R-M				T	
10P-S	T					10R-S				T	

The blank space indicates the part of sequences that are completely matched. Nucleotides were numbered according to reference sequences LC495488.1 (partial sequence of *ATP6* and *cox3*) and AP005995.1 (*D-loop*) of perch and OM736800.1 (*ATP6*) and DQ455036.1 (*D-loop*) of roach.

## Results

The amount and purity of DNA of both species obtained from the scales and mucus are shown in Supplementary Table S1. The total DNA yield extracted from the mucus was lower, ranging from 34.4 ng/µl (1P-M) to 1924 ng/µl (8P-M) as compared with the DNA yield extracted from the scales, ranging from 56.4 ng/µl (3P-S) to 3711 ng/µl (7P-S) of the perch. Similar results were obtained in roaches, the total DNA yield extracted from the mucus was lower, ranging from 10 ng/µl (9R-M) to 2863 ng/µl (1R-M) as compared with that extracted from the scales, ranging from 284 ng/µl (9R-S) to 2939 ng/µl (4R-S). The mean concentration of DNAextracted from the mucus was 714.04 ng/µl (perch) and 758.1 ng/µl (roach) and 1236.24 ng/µl (perch) and 1817.4 ng/µl (roach) DNA extracted from the scales. The purity of DNA extracted from the mucus, determined from the A260/A280 ratio, ranged from 1.248 (9P-M) to 2.156 (1P-M) of the perch, and that of the roach ranged from 1.165 (10R-M) to 1.870 (9R-M). The purity of DNA extracted from the scales, determined from the A260/A280 ratio, was higher as compared with that of DNA extracted from the mucus, and ranged from 1.516 (10P-S) to (2.091) (3P-S) of the perch, and that of the roach ranged from 1.561 (2R-S) to 1.871 (9R-S).

**Figure 1.**
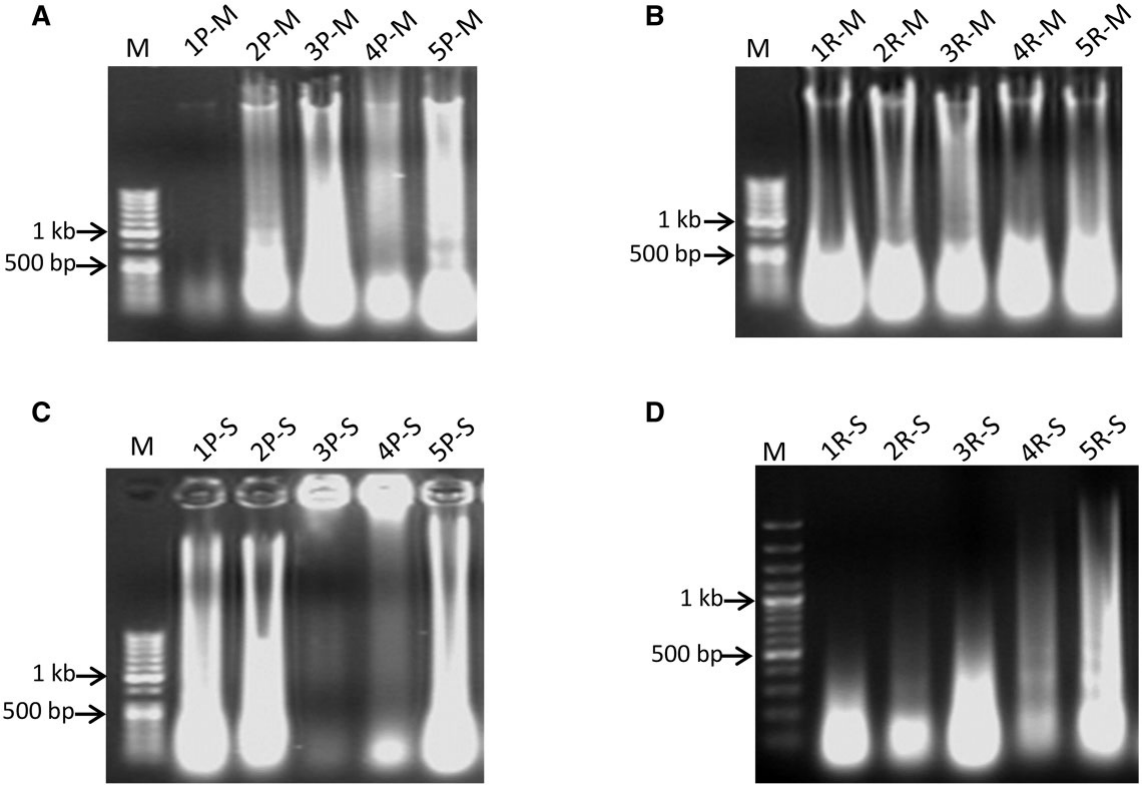
Agarose gel electropherograms demonstrating relative quantity and quality of DNA extracted from the mucus (**A**) of five individuals of perch and (**B**) five individuals of roach, and DNA extracted from the scales (**C**) of five individuals of perch and (**D**) five individuals of roach.

All samples of extracted DNA were subjected to the agarose gel electrophoresis analysis to determine the integrity of DNA (Fig. 1). A substantial level of DNA degradation was observed in all 40 samples. Comparing DNA isolated from the scales and mucus of the same individual, the results of agarose gel electrophoresis revealed a better quality of DNA extracted from the mucus (Fig. 1A and B) as compared to that of DNA obtained from the scale (Fig. 1C and D).

**Figure 2.**
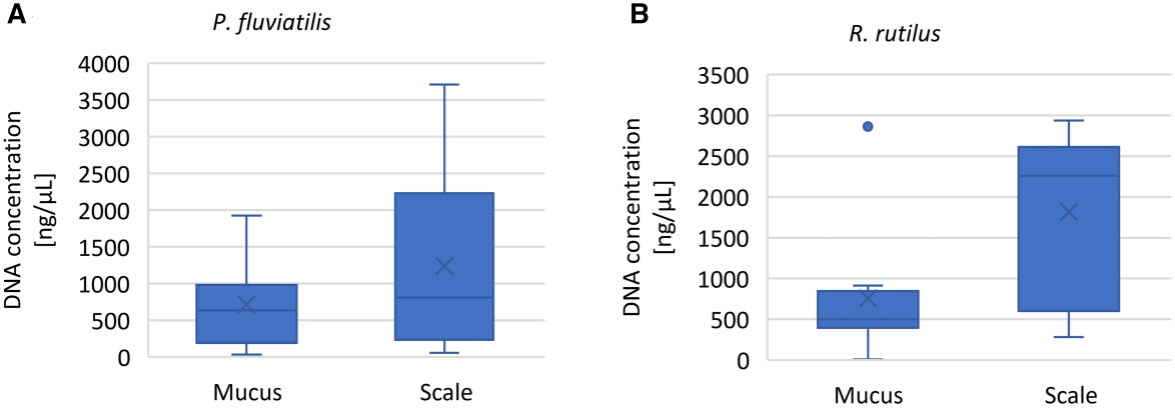
Comparison of the DNA concentration extracted from mucus and scale of perch (**A**) roach (**B**). For each box plot, *x* in the box represents the mean; the line in boxes represents the median; top and bottom lines represent maximum and minimum values of the data, respectively; dots represent outliers.

The results showed a higher yield of DNA extracted from the scales than that of DNA extracted from the mucus in the perch. A significant difference in DNA concentration was observed between DNA extracted from the mucus and scales in the roach (*P* < 0.05; Fig. 2). A higher variation in the DNA concentration from the mucus and scales was detected in the perch as compared to that in the DNA concentration from the roach.

**Figure 3.**
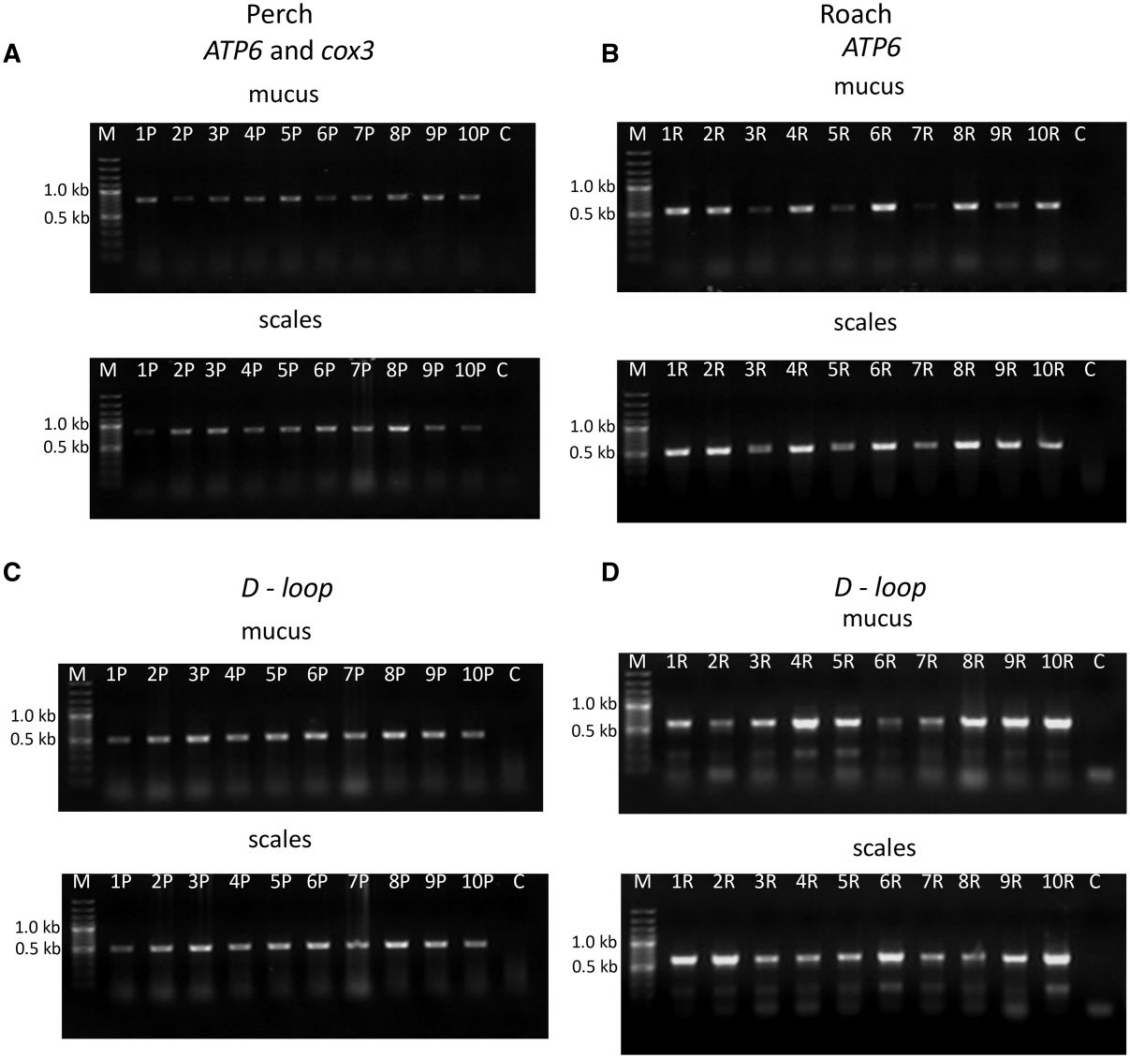
Agarose gel electropherograms of PCR products using mtDNA *ATP6* gene fragment extracted from mucus and scale of (**A**) perch and (**B**) roach. Agarose gel electropherograms of PCR products using mtDNA *D-loop* region extracted from mucus and scale of (**C**) perch and (**D**) roach. M: Marker 100 bp ladder; C: Negative control.

To determine whether DNA extracted from the mucus and scale is suitable for further molecular studies, we amplified mtDNA fragments of the *ATP6* gene (Fig. 3A and B) and the *D-loop* regions (Fig. 3C and D) of the perch and roach. The results showed a high amount and clear bands of PCR products using DNA isolated from the mucus and scales of both fish species studied.

To assess the repeatability of sequencing of mtDNA *ATP6* and *D-loop* fragments, sequences obtained using DNA derived from the mucus and scales were compared across all perch and roach samples studied. The trimmed sequence of *ATP6* fragment included627 bp, and sequences of *D-loop* contained 438 bp for theperch and trimmed sequences of *ATP6* and *D-loop* fragments included 465 bp and 486 bp for the roach, respectively. The amplifiedfragments obtained using DNA extracted from the mucusand scale of the same individual revealed 100% identity inallcases (Table 2). Among 10 specimens studied overall, 2nucleotidesubstitutions compared to the reference sequences were detected in the *ATP6* mtDNA region and 3 ones were determined in *D-loop* sequence of the perch. A single-nucleotide substitution was detected in the *ATP6 gene* region, and four nucleotide substitutions were determined in *D-loop* sequence of the roach as compared to the reference sequences, revealing a potential for the studied mtDNA loci to be used to discover intraspecific genetic variability using the non-invasive DNA extraction method.

## Discussion

The isolation of sufficient DNA quantity and quality collected non-invasively from such samples as fish scales or mucus has become of wide interest to researchers studying genetic diversity [5]. To study the genetic structure of fish populations, fish must be caught, and DNA extracted mainly from the muscle [31] or the fin [32] tissue. Such research is difficult to carry out on a large scale, as it requires the killing of many fish [33] or harming them. However, it was demonstrated that it is possible to extract DNA from the scale of the roach [34] using the CTAB method [35]. DNA extraction from the mucus such as skin swabbing has been used successfully in many fish species including *Neolamprologus pulcher* [36], *Gadus morhua* [37], *Lepomis macrochirus* [38], *Oreochromis niloticus* [39], or zebrafish [17]. So far, there has been a lack of information on whether extracting DNA from the mucus is suitable for studies into a genetic structure of a population due to a higher risk of DNA contamination [18] as compared to DNA obtained from the scales. In this study, we extracted DNA from the scale and mucus of two freshwater fish species perch and roach using the Aljanabi and Martinez [24] method. We selected the least invasive “swabbing/wiping” method to extract DNA from the mucus in both fish species. DNA isolation with the treatment of RNAse can decrease the quantity of DNA and prevent further amplification procedures [40]. In this study, we used a DNA extraction method ensuring triple protection of DNA strands (by EDTA, SDS, and NaCl); this environment is not suitable for RNase seeking to get DNA free of RNA [41]. In this study, RNAse treatment was not given.

The yield and quality of DNA extracted from the mucus and scales have been compared. The agarose gel electrophoresis analysis revealed a higher quality of DNA extracted from the mucus (Fig. 1A and B) as compared to that of DNA obtained from the scales (Fig. 1C and D). A large amount of degraded DNA was detected from both DNA extraction tissues (Fig. 1). DNA degradation and DNA damage occur during enzymatic processes, oxidative damage, UV radiation, and hydrolysis [42]. DNA damage starts immediately after sampling from a live specimen [43] and continues to degrade regardless of the way DNA has been preserved [44]. In this study, DNA was extracted from thawed fish 2 months after capture and freezing, which could have had an impact on DNA degradation. Also, a large amount of DNA may be degraded in the homogenizing buffer. Of course, some loss of DNA is often acceptable for further applications but only if this loss does not affect the quality and quantity of the sample. In our case, we showed that extracted DNA can be used for PCR amplification and sequencing. A higher concentration of DNA was detected in scales, which could have been due to a greater weight of the sampled tissue as compared to the samples of the mucus (Fig. 2). DNA extracted from the mucus showed a relatively lower purity, as compared to DNA extracted from the scales (Supplementary Table S1), indicating the presence of a larger number of proteins, phenol, or other contaminants [45]; however, in most cases, it was not beyond the acceptable range. In general, the ratio of A260/A280 indicated acceptable purity between 1.6 and 2.0 [46, 47].

Unlike nuclear DNA, mtDNA is known to have a higher rate of nucleotide substitution [48], and the analysis of mitochondrial genetic diversity is widely used in population genetics. To determine whether DNA extracted from the mucus or scales is suitable for a nucleotide sequencing analysis, we amplified mtDNA fragments from both the coding region (*ATP6* gene fragment) and the non-coding one (*D-loop*). Here, we developed primers for two mtDNA markers including the *ATP6* gene region and *D-loop* region of the roach. The results showed that DNA isolated from the mucus and scales is sufficient for PCR amplification (Fig. 3). The DNA sequence alignment analysis revealed identical sequences attributed to the same individual extracted from two different areas (scales and mucus) (Table 2) showing no DNA contamination.

We have demonstrated that skin swabbing can be used to collect a sufficient amount of DNA for PCR amplification and nucleotide sequence analysis. In this article, we provided a detailed protocol for DNA extraction from the scale and mucus using skin swabbing to obtain high-quality DNA from freshwater fish such as the roach and perch. The procedure is quite simple, rapid and can be completed at a lower cost than using a commercial kit. The method described is less invasive and does not require anesthetizing or euthanizing the fish and ensures the possibility of releasing it back into the water where it was caught in case you work with live fish.

## Conclusion

While higher DNA concentrations were obtained from the scales, the collection of scales may cause some stress to fish. A smaller quantity and higher quality of DNA yield were extracted from the mucus. In carrying out the DNA sequencing analysis, we revealed that by using the swabbing method, it was possible to obtain high-quality DNA. Considering the degree of invasiveness, we propose mucus as biological material for DNA extraction in both fish species for further studies. In the present article, we compared the efficiency of the method used for DNA extraction, amplification, and sequencing based on DNA obtained from perch and roach collected from the scales and mucus, which provided evidence that the results of the quantity and quality of extracted DNA, as well amplification and sequencing of different mtDNA fragments, were reproducible and sufficient to be applied in the studies of genetic variability of different fish species.

## Authors’ contributions

Ieva Ignatavičienė (Conceptualization [equal], Data curation [lead], Formal analysis [equal], Investigation [lead], Methodology [lead], Project administration [equal], Software [equal], Validation [equal], Visualization [equal], Writing—original draft [lead], Writing—review and editing [equal]), Adomas Ragauskas (Conceptualization [equal], Data curation [equal], Methodology [equal], Validation [equal], Writing—review and editing [equal]), Vytautas Rakauskas (Resources [lead], Validation [equal], Writing—review and editing [equal]), and Dalius Butkauskas (Conceptualization [equal], Data curation [equal], Formal analysis [equal], Funding acquisition [lead], Investigation [equal], Methodology [equal], Project administration [lead], Supervision [lead], Validation [equal], Visualization [equal], Writing—original draft [equal], Writing—review and editing [equal]).

## Supplementary Material

bpad022_Supplementary_Data
