# Supplementary material for: Quality of DNA extracted from freshwater fish scales and mucus and its application in genetic diversity studies of Perca fluviatilis and Rutilus rutilus
Source: Biol Methods Protoc. 2023 Sep 28;8(1):bpad022. doi: 10.1093/biomethods/bpad022 (PMC10561993; doi:10.1093/biomethods/bpad022)
Supplement: bpad022_Supplementary_Data [file bpad022_supplementary_data.docx]

**Manuscript Number: BMP-2023-033.R2**

**Supplementary data**

**Supplementary Table 1:** DNA concentration of perch and roach extracted from mucus and scale.

| **Sample of perch** | **Tissue from which DNA was isolated** | **ng/µL** | **A260/A280** |  | **Sample of roach** | **Tissue from which DNA was isolated** | **ng/µL** | **A260/A280** |
| --- | --- | --- | --- | --- | --- | --- | --- | --- |
| 1P-M | mucus | 34.4 | 2.156 |  | 1R-M | mucus | 2863 | 1.598 |
| 2P-M | mucus | 176 | 1.972 |  | 2R-M | mucus | 914 | 1.776 |
| 3P-M | mucus | 538 | 1.976 |  | 3R-M | mucus | 500 | 1.283 |
| 4P-M | mucus | 197 | 2.100 |  | 4R-M | mucus | 821 | 1.489 |
| 5P-M | mucus | 500 | 2.018 |  | 5R-M | mucus | 414 | 1.216 |
| 6P-M | mucus | 759 | 1.827 |  | 6R-M | mucus | 505 | 1.515 |
| 7P-M | mucus | 824 | 1.836 |  | 7R-M | mucus | 471 | 1.208 |
| 8P-M | mucus | 1924 | 1.470 |  | 8R-M | mucus | 346 | 1.831 |
| 9P-M | mucus | 1453 | 1.248 |  | 9R-M | mucus | 10 | 1.870 |
| 10P-M | mucus | 735 | 1.935 |  | 10R-M | mucus | 737 | 1.165 |
| **Mean** | | **714.04** | **1.854** |  |  | **Mean** | **758.1** | **1.495** |
| 1P-S | scale | 2056 | 1.916 |  | 1R-S | scale | 2576 | 1.635 |
| 2P-S | scale | 1176 | 1.920 |  | 2R-S | scale | 2211 | 1.561 |
| 3P-S | scale | 56.4 | 2.091 |  | 3R-S | scale | 2306 | 1.625 |
| 4P-S | scale | 211 | 1.911 |  | 4R-S | scale | 2939 | 1.597 |
| 5P-S | scale | 934 | 1.964 |  | 5R-S | scale | 2728 | 1.745 |
| 6P-S | scale | 539 | 1.897 |  | 6R-S | scale | 1490 | 1.551 |
| 7P-S | scale | 3711 | 1.951 |  | 7R-S | scale | 2471 | 1.428 |
| 8P-S | scale | 684 | 1.951 |  | 8R-S | scale | 551 | 1.744 |
| 9P-S | scale | 2757 | 1.963 |  | 9R-S | scale | 284 | 1.871 |
| 10P-S | scale | 238 | 1.516 |  | 10R-S | scale | 618 | 1.826 |
| **Mean** | | **1236.24** | **1.908** |  |  | **Mean** | **1817.4** | **1.658** |
